# Methods for tuning plasmonic and photonic optical resonances in high surface area porous electrodes

**DOI:** 10.1038/s41598-021-86813-y

**Published:** 2021-04-07

**Authors:** Lauren M. Otto, E. Ashley Gaulding, Christopher T. Chen, Tevye R. Kuykendall, Aeron T. Hammack, Francesca M. Toma, D. Frank Ogletree, Shaul Aloni, Bethanie J. H. Stadler, Adam M. Schwartzberg

**Affiliations:** 1 Department of Electrical and Computer Engineering, University of Minnesota, Minneapolis, USA; 2 The Molecular Foundry, Lawrence Berkeley National Laboratory, Berkeley, USA; 3 Joint Center for Artificial Photosynthesis, Lawrence Berkeley National Laboratory, Berkeley, USA; 4 Chemical Sciences Division, Lawrence Berkeley National Laboratory, Berkeley, USA

**Keywords:** Optical materials and structures, Materials for devices, Materials for energy and catalysis, Materials for optics, Energy, Thermoelectric devices and materials, Electrical and electronic engineering

## Abstract

Surface plasmons have found a wide range of applications in plasmonic and nanophotonic devices. The combination of plasmonics with three-dimensional photonic crystals has enormous potential for the efficient localization of light in high surface area photoelectrodes. However, the metals traditionally used for plasmonics are difficult to form into three-dimensional periodic structures and have limited optical penetration depth at operational frequencies, which limits their use in nanofabricated photonic crystal devices. The recent decade has seen an expansion of the plasmonic material portfolio into conducting ceramics, driven by their potential for improved stability, and their conformal growth via atomic layer deposition has been established. In this work, we have created three-dimensional photonic crystals with an ultrathin plasmonic titanium nitride coating that preserves photonic activity. Plasmonic titanium nitride enhances optical fields within the photonic electrode while maintaining sufficient light penetration. Additionally, we show that post-growth annealing can tune the plasmonic resonance of titanium nitride to overlap with the photonic resonance, potentially enabling coupled-phenomena applications for these three-dimensional nanophotonic systems. Through characterization of the tuning knobs of bead size, deposition temperature and cycle count, and annealing conditions, we can create an electrically- and plasmonically-active photonic crystal as-desired for a particular application of choice.

## Introduction

The term “plasmonics” was initially coined as an analogous term to “photonics” and “electronics”, after early researchers realized that surface plasmons could enable a new class of devices based on their unique properties^1,2^. Surface plasmons are electron density waves that are bound to the interface of a metallic and a dielectric material and are induced by incoming resonant photons. Surface plasmons generate strong, high-gradient electric fields that are particularly sensitive to the surrounding media and have been applied to circuits and inter-device communications^3,4^, biological- and chemical-sensing and imaging^5–7^, precision light sources^8–10^, photovoltaics^11,12^, CO_2_ reduction^13–16^, solar water splitting^17–20^, and as nanoscale heaters for heat-assisted magnetic recording data storage^21,22^.

To investigate how plasmons interact with other photonic and electronic phenomena in three-dimensional architectures^23,24^, we began investigating high surface area, plasmonically-active photonic crystal electrodes. Similar to the way periodic atomic arrangement controls electron propagation, periodic nanostructured features can be used to transmit or block certain light frequencies in photonic crystals^25,26^. Photonic crystals have enabled many advanced technologies including microlasers^27^, light emitting diodes^28^, dye-sensitized solar cells^29^, and functional built-in elements and optical fibers for telecommunications^30,31^. Templated opal and inverse opal structures are frequently used in photonic crystal applications due to their relative ease of fabrication^32–34^. However, creating high surface area photonic crystals with conducting surfaces is challenging. Many applications can benefit from such structures, including emitters for electrically tunable color films^34–36^ and thermophotovoltaics^37–39^ as well as high surface area electrodes for solar water splitting^17,40^ and supercapacitors^41,42^. Furthermore, combining the properties of both photonic light trapping and plasmonic field enhancement in a single material, it may be possible to increase the efficiency of a variety of the aforementioned optically driven processes including light harvesting materials and devices. A major challenge, however, is the large increase in absorption and reflection that occur as plasmonic effects increase. This phenomenon may dampen or destroy the photonic resonance, negating any cooperative effects. In some cases, conductive and plasmonic materials like silver may enable the desired quality of photonic crystals^43^; however, creating such complex metallic photonic crystals is challenging because noble metals are soft and mechanically unstable, whereas hard metals are reactive, and forming continuous ultrathin (< 7 nm) metal layers into three-dimensional structures is difficult to achieve.

TiN is a highly conductive and plasmonically active ceramic with excellent chemical and mechanical stability^44,45^. For over 20 years, TiN has been used for conformal and three-dimensional ultrathin conducting films using thermal atomic layer deposition (ALD) and plasma-enhanced ALD (PEALD) at the industrial scale^46,47^. Despite a long history of application of ALD and PEALD to creating or coating photonic crystals with a diverse variety of high quality materials over complex geometries, ALD TiN-based three-dimensional conductive photonic crystals have not been explored to the best of our knowledge^48–53^.

In this work, we demonstrate the ability to produce high surface area, porous, bicontinuous, three-dimensional, photonic crystal structures that are capable of trapping and controlling light using conductive, plasmonic TiN conformally coated onto a SiO_2_ photonic crystal by PEALD. Conformal coatings of TiN as thin as 2 nm were used to study the plasmonic, photonic, and electronic properties of an ultrathin film applied to a non-conducting photonic crystal. The TiN-coated structure is both a conducting photonic crystal and a high surface area electrode with possible applications utilizing the plasmonic, photonic, and electronic effects. We use thermal treatments to demonstrate simultaneous tuning of the plasmonic, photonic, and ohmic response.

## Results and discussion

**Figure 1:**
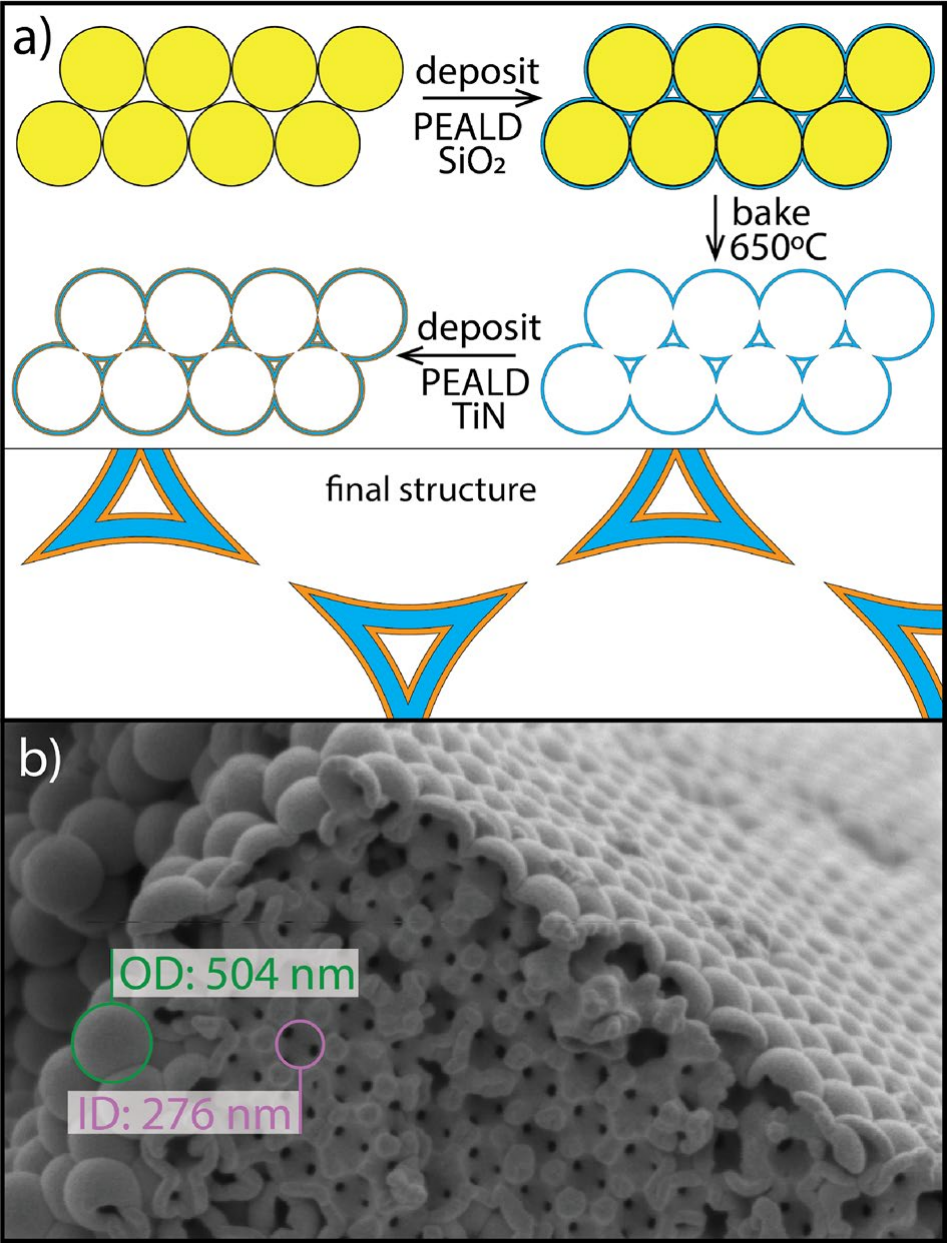
Inverse opal fabrication. Fabrication of TiN plasmonic, photonic, and conductive inverse opals. (**a**) Polystyrene beads in solution were evaporated onto a quartz slide and allowed to dry. These opals were partially infilled with a conformal layer of PEALD SiO_2_ and then the organic component was removed by annealing. The remaining SiO_2_ inverse opal was then partially infilled with a conformal layer of PEALD TiN. (**b**) A post-anneal scanning electron micrograph of an inverse opal coated with 47 nm of TiN shows the complex, periodic, and high surface area conductive inverse opal lattice. Starting with a 390 nm polystyrene sphere, a 10 nm SiO_2_ conformal coating and a 47 nm TiN conformal coating will yield an outer diameter of 504 nm (in green) and an inner diameter of 276 nm (in purple).

Conventionally, inverse opal structures are formed by *fully* infilling the void space in self-assembled opal templates, followed by removal of the template. In order to produce extremely high surface area electrodes with both photonic and plasmonic properties, we have developed a *partially* infilled inverse opal, effectively doubling the surface area over conventional inverse opals, layered with a conductive and plasmonic TiN thin film that coats all surfaces. For simplicity, we refer to this structure as an inverse opal. The fabrication process and resulting structure are shown in Fig. 1 following the initial self-assembly of a polystyrene opal template as previously demonstrated^33^. In principle, one could coat this opal with PEALD grown TiN and obtain a high surface area, plasmonic, photonic crystal electrode; however, the polystyrene template is unstable above 100 °C due to close proximity to the polymer-glass transition, and TiN grown below this temperature is relatively low quality. In addition, extremely thin TiN films will be necessary in order to obtain the required optical transmission, such that structural stability would likely be compromised. We are able to overcome all of these challenges by first coating the opal template with silica, followed by removal of the polystyrene template by annealing in air, and finally performing PEALD of TiN on the SiO_2_ template at temperatures near 300 °C. The result is a low density, inverse opal structure with significantly enhanced surface area. Figure 1b shows a scanning electron microscopy (SEM) image of a sample with a 47 nm TiN coating.

**Figure 2:**
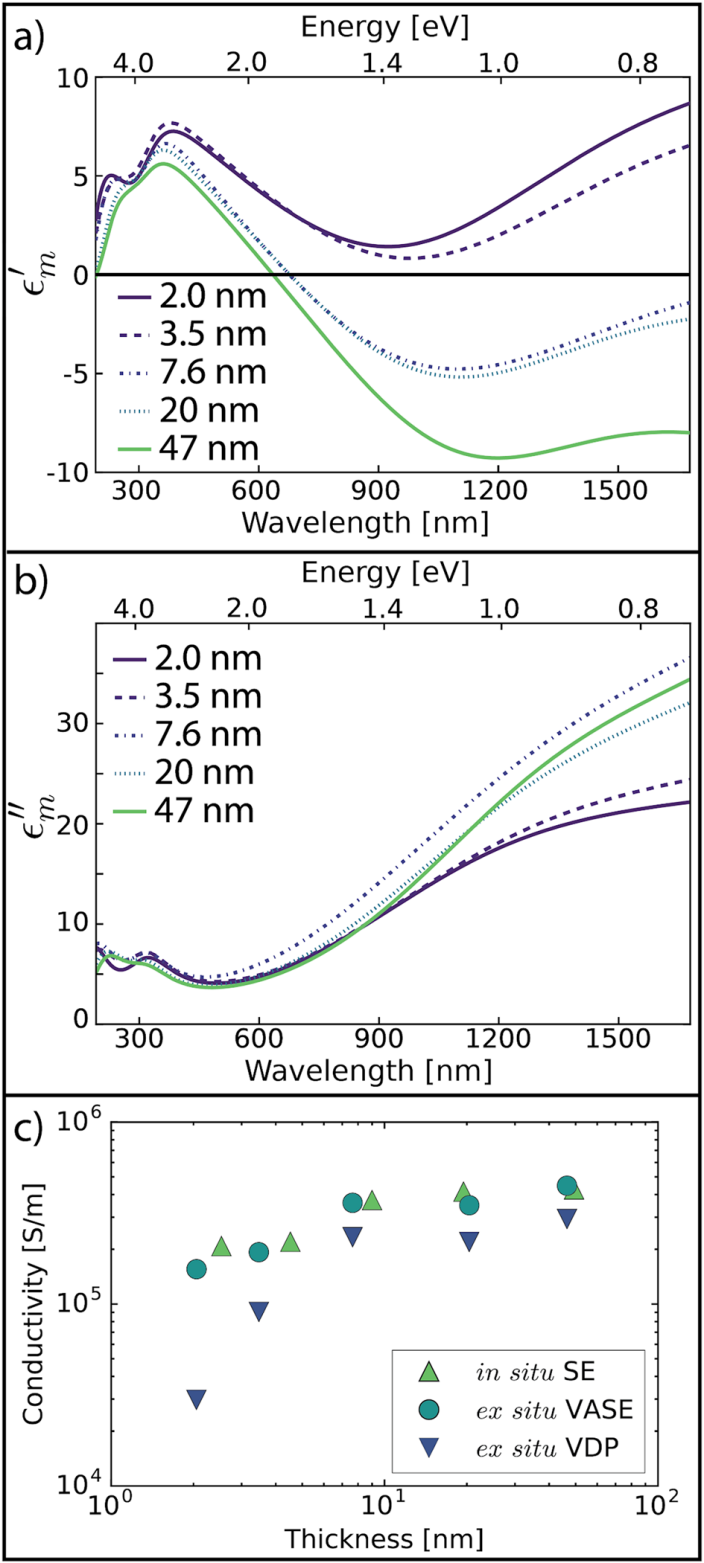
Dielectric function and conductivity. Optical and electronic properties of TiN thin films deposited on SiO_2_-coated silicon substrates. The real (**a**) and imaginary (**b**) parts of the dielectric function fit to ex situ VASE data for each TiN film thickness (2–47 nm). (**c**) Spectroscopic ellipsometry was also used to measure the film thickness and conductivity both in situ (green upright triangle) and after deposition and exposure to air using the VASE method (blue circle). These optical measurements of conductivity are compared to electrical transport measurements performed using the van der Pauw (VDP) method (inverted purple triangle).

To explore the optical and electronic quality of the as-deposited TiN, we used in situ spectroscopic ellipsometry (SE) to monitor the growth process on planar substrates (silicon with 250 nm of thermally grown SiO_2_). Ex situ variable-angle SE (VASE, supplementary Figure S1) was used for more detailed analysis of air-exposed samples, and van der Pauw measurements were used to directly probe the conductivity of films deposited on the planar substrates. Ex situ VASE data was acquired at 5 degree intervals between 45 and 75 degrees and analyzed using a parametric fit to a Drude-Lorentz model from which we extract the films’ dielectric function and conductivity^54–56^. The best fit was achieved by including an approximately 1.5 nm thick TiO_2_ layer and correspondingly reduced TiN thickness. This native oxide layer forms upon exposure to air and is not observed in the in situ SE measurements. Figure 2a,b show the real and imaginary parts, respectively, of the dielectric function obtained by ex situ VASE of films ranging from 2 to 47 nm (all thicknesses measured by ex situ VASE). The 2.0 and 3.5 nm thick films do not demonstrate plasmonic behavior as ${\epsilon }_{m}^{^{\prime}}$ is positive across all measured wavelengths. A transition to plasmonic behavior is observed for thicknesses above 3.5 nm. The 7.6 nm thick TiN film becomes weakly plasmonic (${\epsilon }_{m}^{^{\prime}}$ is negative) for much of the red and near infrared portions of the spectrum. The lack of plasmonic behavior in ultrathin (< 7 nm) films is likely a result of lower conductivity (discussed below) due to reduced grain size and oxidation at nanocrystalline grain boundaries, which becomes less significant as the film thickness increases^57^. In situ SE measurements suggest that even the thinnest TiN films showed modest plasmonic behavior as grown (supplementary Figure S2); however, their plasmonic properties were significantly degraded upon exposure to air, presumably due to a reduction in free carrier density from film oxidation.

Interestingly, the thinnest films produced for this study still show a technologically useful level of conductivity^36^, as shown by the VASE and van der Pauw measurements in Fig. 2c. This implies that even though the plasmonic behavior of these films is destroyed upon exposure to oxygen^54,55,57^, a number of conductive pathways exist at thicknesses close to the native oxide thickness (~ 1–2 nm). Post-oxidation conductivity saturates for TiN films thicker than ~ 10 nm. This is a critically important factor when considering high surface area electrodes, which will require extremely thin films to maintain optical activity due to the inherent opacity of plasmonic TiN films. We are able to use these properties to fabricate high-porosity, transparent, conductive electrodes that are stable under ambient conditions. Furthermore, it is possible that a layer of SiN_x_ could be used to passivate the TiN surface against oxidation and further enhance its ex situ properties. Finally, the van der Pauw measurements universally show slightly lower conductivity than the SE or VASE measurements. As a direct electrical measurement, the van der Pauw conductivity is strongly affected by grain boundary scattering, which will be significant in these nanocrystalline films. On the other hand, SE and VASE are all-optical measurements, and hence are relatively insensitive to grain boundary scattering of carriers. As such, this difference is more significant in thinner films with smaller grains.

**Figure 3:**
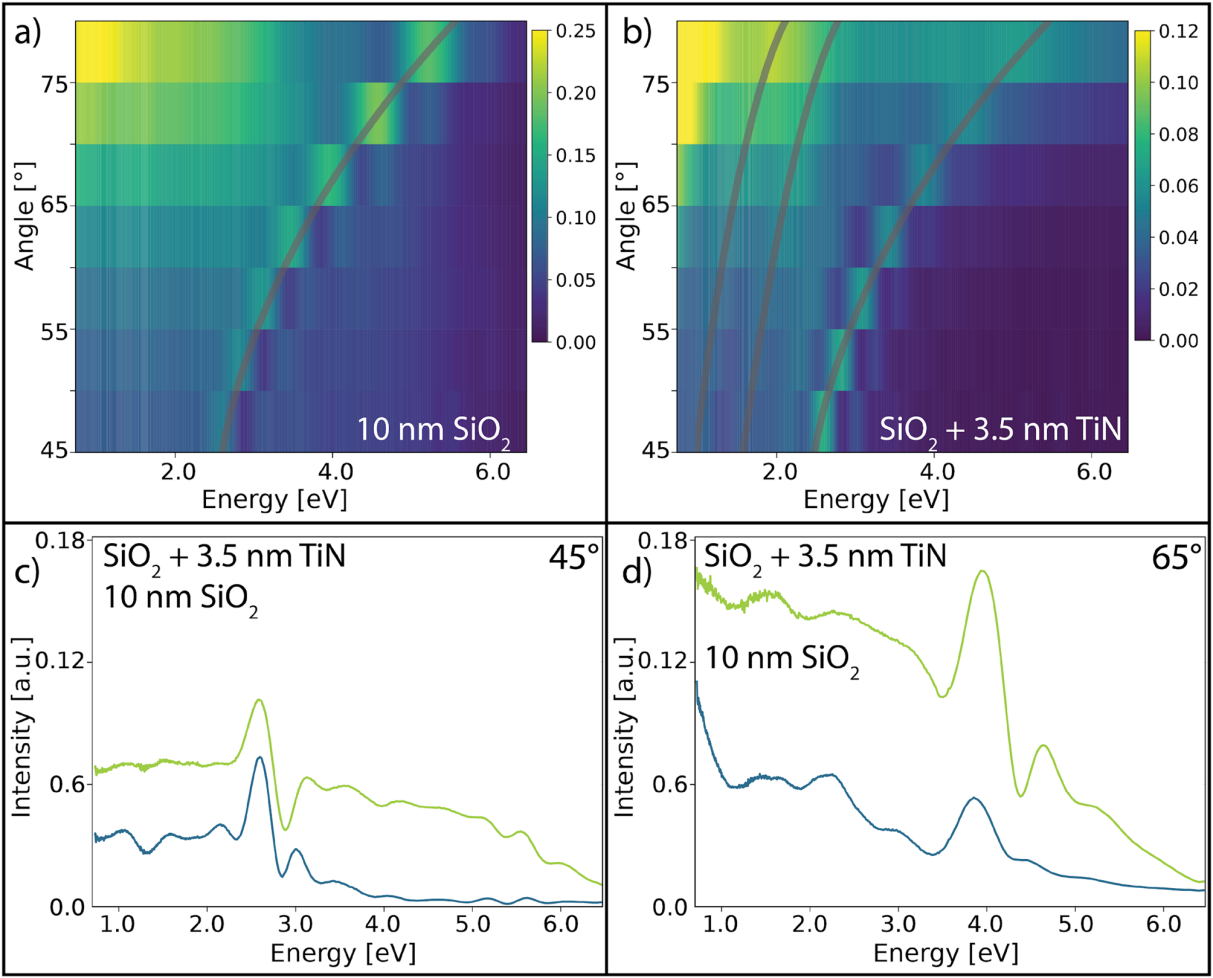
Inverse opal reflected intensity. Reflected intensity measured by VASE. (**a**) A strong photonic mode is present in the 10 nm SiO_2_ inverse opal, which (**b**) is somewhat attenuated after coating with 3.5 nm of conductive and refractory TiN. Two additional weak low energy modes can be seen in (**b**) after TiN coating. Comparison of the reflected intensity of two inverse opal samples measured at (**c**) 45° and (**d**) 65°. A small red-shift in photonic resonance after TiN deposition is visible at 65°.

With an understanding of the optical and electronic properties of the planar TiN thin films, we investigated the properties of the SiO_2_ and SiO_2_/TiN inverse opals. Using variable-angle reflectometry (VAR), we were able to probe the angle-dependent optical properties of the bare SiO_2_ scaffold and the 3.5 nm TiN-coated structures, shown in Fig. 3a,b, respectively. In both structures, the dominant resonance shifts from approximately 2.5 eV to 5 eV as the measurement angle increases from 45° to 80°, which is traced by gray lines. This shift is expected for opal or inverse opal structures, as the structure periodicity, which determines the optical response, varies as a function of the incident beam angle^34,58^. It is of particular note that the dominant photonic resonance is maintained in the TiN coated structure despite the presence of the conductive and plasmonic coating. The main modifications to the photonic response are a slight damping of the mode intensity and the appearance of two additional weak resonances at low energy (also traced by gray lines in Fig. 3b), likely due to new optical modes originating from the TiN coating. Examining individual spectra reveals the extent of modification induced by the TiN film. Figure 3c shows the reflected intensity of the SiO_2_ and SiO_2_/TiN structures at 45°, where a small shift in the main resonance (~ 50 meV) has taken place, but the spectrum is largely unchanged. Figure 3d shows that the resonance shift (~ 140 meV) becomes more significant at 65°, and resonance damping is clear, with ~ 50% reduction in intensity. Increased effects from the presence of the TiN thin film are expected at glancing angles as the reflected light path length within the material increases, and therefore the number of SiO_2_/TiN/air interfaces, increases. At these angles, where the light passes through the most material, the photonic response is relatively unperturbed by the presence of the conductive TiN film. This property implies a general usefulness to this technique that will allow for the conversion of any porous photonic structure into a conductive electrode, with minimal impact on optical properties.

In order to understand the photonic-plasmonic interactions within the SiO_2_/TiN inverse opal structure, we compare the plasmonic resonance of the planar TiN films (which are not photonic and therefore decoupled) to the SiO_2_/TiN inverse opal photonic resonance. The plasmonic figure of merit (FOM) expresses the energy-dependent quality factor of the plasmonic resonance and is defined as the ratio of the real and imaginary part of the dielectric function (FOM = $-{\epsilon }_m^\prime$/${\epsilon }_m^{\prime\prime}$). The plasmonic transition energy (E_pl_, where ${\epsilon }_m^\prime=0$ and becomes non-negative) is the high-energy cutoff of the plasmonic response. As E_pl_ moves towards and overlaps with the photonic resonance, there is the potential for maximizing both optical effects.

**Figure 4:**
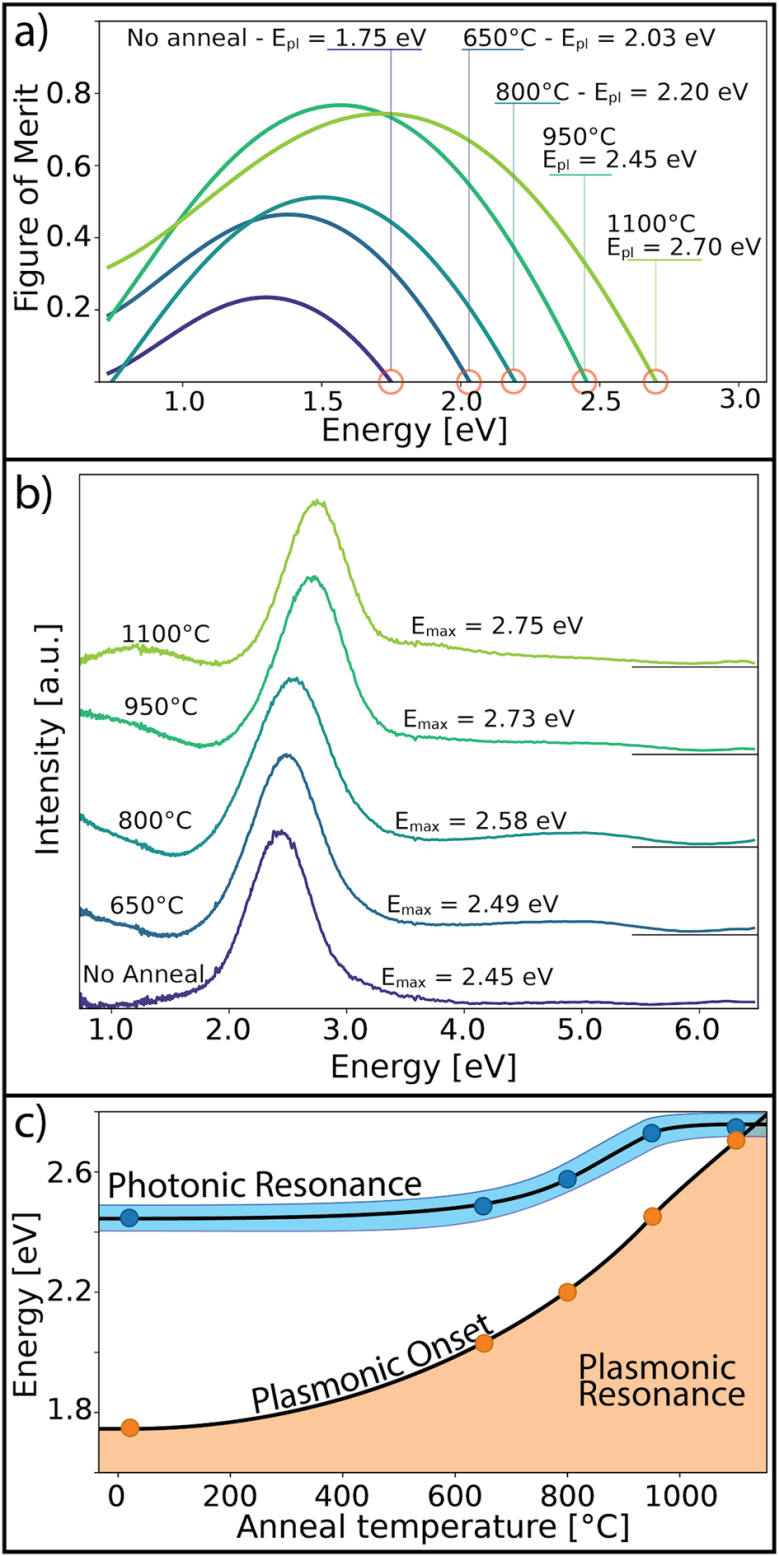
Annealing effects on planar TiN films and inverse opal samples. Annealing changes the plasmonic properties of the TiN. (**a**) The figures of merit (ratio of the real and imaginary parts of the dielectric function where the real part is negative) after each annealing temperature on a 150-cycle two-dimensional planar film demonstrate the evolution in film plasmonic quality in the visible and near infrared. The plasmonic transition energies (E_pl_) of the films blue-shift with increasing annealing temperature. (**b**) The SiO_2_/TiN photonic crystal resonance peak also blue-shifts with increasing annealing temperature, though to a lesser degree. (**c**) Overlapping the two trends—photonic and plasmonic resonance—show that with the right material properties, plasmonically and photonically active electrodes can be designed.

In Fig. 4a, we show the FOM of a TiN sample (~ 7.6 nm thick) before and after thermal annealing at different temperatures. These measurements were taken on planar substrates that were deposited and annealed concurrently with the photonic structures. The non-annealed sample has an E_pl_ of 1.75 eV, placing it far from the photonic resonance position (E_max_) of this structure at 2.45 eV, which is shown in Fig. 4b. By annealing the TiN films under a reducing atmosphere, we were able to modify and improve the plasmonic response of even extremely thin TiN films. In this case, annealing enhanced the FOM and blue-shifted E_pl_ closer to the photonic resonance. One would expect an increased plasmonic FOM to result in damping of the photonic resonance in the SiO_2_/TiN structure due increased reflectivity. However, upon annealing at 1100 °C, we see a large increase in the plasmonic FOM with minimal damping of the photonic resonance. Additionally, we observe a shift of E_pl_ to 2.70 eV (∆0.95 eV) in the planar TiN films (Fig. 4a), but a less significant change to E_max_, now at 2.75 eV (∆0.3 eV), in the SiO_2_/TiN structure (Fig. 4b). In Fig. 4c, we directly compare the effect of annealing on E_pl_ and plasmonic resonance of the planar TiN films to the photonic resonance of the SiO_2_/TiN inverse opal structure. Both curves show a clear blue-shift upon annealing; however, the effect is more significant for E_pl_ than E_max_, likely due to crystallinity and purity improvement in the TiN from annealing. The shift in E_max_ is likely due to densification of both the SiO_2_ and TiN, which induces small structural and dielectric response changes. Despite the likely structural changes taking place under these conditions, the thin film inverse opal with wall thicknesses of 25 nm (TiN/SiO_2_/TiN—7.6/10/7.6 nm) and a height of approximately 4 microns (~ 10 layers of 390 nm beads) remains stable up to 1100 °C. This allowed us to tune the plasmonic and photonic resonances into an overlapping regime with little, if any, degradation of optical properties of the photonic structure. This is a significant result because it implies that it is possible to decouple the plasmonic and photonic interactions within the SiO_2_/TiN inverse opal structure, enabling a highly porous, transparent electrode structure where the light trapped by the photonic structure can be paired with the plasmonic resonance energy of the TiN coating without damping effects.

In summary, we have demonstrated the ability to fabricate three-dimensional, plasmonically active photonic crystals using PEALD of TiN in an inverse opal-like structure. The TiN plasmonic resonance can be tuned via thermal treatment to be near the photonic resonance of the entire structure, allowing for overlapping optical modes to be present, which can allow one to take advantage of both photonic light guiding and plasmonic resonance enhancement without sacrificing the optical properties of either. We used planar TiN films, VASE, and van der Pauw measurements to provide insight into the electronic and plasmonic properties of the TiN-coated photonic crystal structures. Using different sized polystyrene beads as a template^34^ and other ALD film thicknesses to form the inverse opal could result in additional tuning of the plasmonic and photonic resonance overlap. The methods demonstrated in this work could be extended to more complex template structures, including three-dimensional chiral helices, in an industrially compatible manner using holographic lithography^59–63^. We believe such structures discussed herein could double as high surface area photoelectrodes for additional electronic functionality and find use in renewable energy systems and many other applications.

## Materials and methods

Inverse opals were grown through a multi-step assembly and coating process. First, self-assembled polystyrene (PS) opal structures were formed by evaporating colloidal solutions of PS beads onto quartz substrates using previously reported methods^33^ and as described below.

### Polystyrene opal synthesis

Substrates (quartz, 1 mm thick, purchased from VWR) were cleaved into 1 × 2 cm^2^ pieces and were cleaned in a three-step process: first, a 15-min sonication in an Alconox/deionized (DI) water solution, second, a 15-min sonication in pure DI water, and third, a 15-min sonication in a 1:1:1 acetone:ethanol:DI water solution. This cleaning process was followed by a 5-min plasma treatment in air. Substrates were inspected between each step, and dust particles were removed with a flow of N_2_.

The slow-evaporation deposition of the PS bead opal templates is described in detail in our publication^33^ and also in an author’s dissertation^64^. To summarize, 390 nm PS bead solutions (non-functionalized, 25 mg/mL in DI water, Bangs Labs) were diluted to 0.1 v/v% using Millipore R water (18.2 MΩcm, 25 °C). Then, a few drops of a 1% solution of TritonX were added (3 drops/8 mL). The prepared substrates were each placed in a small vial (1.5 cm diameter and 4.5 cm height) tilted at a 60º angle (where 0° is horizontal). 16 vials were arranged in a 4 × 4 grid on a hotplate. A 15-min sonication of the prepared PS bead solution was performed just before deposition onto the substrate. Then, 1.5 mL of the prepared PS bead solution were carefully dispensed into each vial. A crystallization dish (15.0 cm diameter and 7.5 cm height) was added to cover the hotplate, and filter paper was included in the bottom of the dish (now above the vials) to absorb and disperse condensation rather than allowing it to drip into the vials. The hotplate was then set to 50 °C, and the humidity and evaporation rate were reproduced by always keeping a consistent amount of water (equal number of filled vials) in the enclosed deposition system. Once the deposition was complete, a 1-hr immersion in ethanol was performed to improve the crystallinity of the periodic aggregate samples. The samples were then carefully dried from the backside using a gentle flow of N_2_. To finish the samples, a 10-min anneal was performed on a hotplate at 90 °C.

### Inverse opal synthesis

Once the PS opals were fully dry, they were conformally coated with 10 nm of SiO_2_ using PEALD at 40 °C using an Oxford Instruments FlexAL PEALD system (tris(dimethylamino)silane precursor, Sigma Aldrich). To remove the PS scaffold the samples were annealed at 650 °C for 30 min in air inside a glass tube furnace (ThermoFisher Scientific Lindberg Blue M, 1" tube) to vaporize the PS, leaving behind the skeletal SiO_2_ structure, which maintained the original morphology. TiN was then deposited conformally onto the scaffold structure via PEALD at 300 °C in thicknesses ranging from ~ 2 nm to 50 nm (tetrakis(dimethylamido)titanium precursor, Sigma Aldrich and a two-stage N_2_ then H_2_ plasma) using another previously reported recipe^56^, resulting in highly porous photonic structures which maintain the original PS structure, as shown in Fig. 1b. TiN growth was monitored via in situ spectroscopic ellipsometry (J. A. Woollam Inc. M2000, 190–1690 nm or 0.73 to 6.9 eV.) on planar reference samples. Ex situ optical properties of the planar TiN films and inverse opals were measured via VASE and VAR (J. A. Woollam Inc. VASE M2000, 190–1690 nm or 0.73 to 6.9 eV.) and at angles ranging from 45° to 75° and 45° to 80°, respectively, in 5° increments. Measurements were analyzed using the CompleteEASE software package (J. A. Woollam Inc.). Conductivity of the planar TiN films was directly measured by the van der Pauw method (Ecopia HMS-5000) at room temperature.

### Annealing of inverse opals and TiN films

This process is also described in an author’s dissertation^64^. A quartz tube furnace (ThermoFisher Scientific Lindberg Blue M, 1" tube, 1100 °C max) was used to perform anneals. Isopropyl alcohol was used to clean the quartz tube at its ends, the sealing attachments, and the quartz slide sample holder. Once the system was pumped to 1.5 × 10^–1^ Torr, a leak check was performed by isolating the pump from the tube and confirming the tube's connections to the upstream gas flow did not have significant leaks. The system was pump-purged (to 1.5 × 10^–1^ Torr, then filled with Ar) twice. 250 sccm Ar and 20 sccm H_2_ were flowed through the Ar-filled tube. Over 10′s of minutes, the furnace temperature was ramped from room temperature (~ 20 °C) to the annealing temperature as fast as possible, followed by a 1-hr anneal, and then cooled. When the samples were cool enough to remove from the furnace (≤ 80–100 °C), the H_2_ gas flow was stopped, and the Ar was allowed to purge the system. Varying TiN coating thicknesses were annealed simultaneously under common conditions and always consistently ordered along the direction of gas flow through the tube with the thinnest samples (planar films on SiO_2_/Si) upstream from the thickest samples (inverse opals on quartz) in order to prevent H_2_ flow shadowing over downstream samples.

Further details of both sample preparation and optical characterization are provided in the supplementary information.

## Supplementary Information

Supplementary Information
