# Supplementary material for: Methods for tuning plasmonic and photonic optical resonances in high surface area porous electrodes
Source: Sci Rep. 2021 Apr 7;11:7656. doi: 10.1038/s41598-021-86813-y (PMC8027385; doi:10.1038/s41598-021-86813-y)
Supplement: Supplementary file 1 — Supplementary Information [file 41598_2021_86813_MOESM1_ESM.docx]

Supplementary information for:

**Methods for tuning plasmonic and photonic optical resonances in high surface area porous electrodes**

Lauren M. Otto^1,2^, E. Ashley Gaulding^3,4^, Christopher T. Chen^2^, Tevye R. Kuykendall^2^, Aeron T. Hammack^2^, Francesca M. Toma^3,4^, D. Frank Ogletree^2^, Shaul Aloni^2^, Bethanie J. H. Stadler^1^, Adam M. Schwartzberg^2,^*

^1^Department of Electrical and Computer Engineering at University of Minnesota

^2^Molecular Foundry at Lawrence Berkeley National Laboratory

^3^Joint Center for Artificial Photosynthesis at Lawrence Berkeley National Laboratory

^4^Chemical Sciences Division at Lawrence Berkeley National Laboratory

*E-mail address: ams@lbl.gov

1. **Spectroscopic ellipsometry and reflectometry**

*In situ* SE (J. A. Woollam M2000) was performed during PEALD SiO_2_ and TiN film deposition at 70º (fixed) and offers the advantages of following the film’s properties during growth and before exposure to outside air leading to surface oxidation.


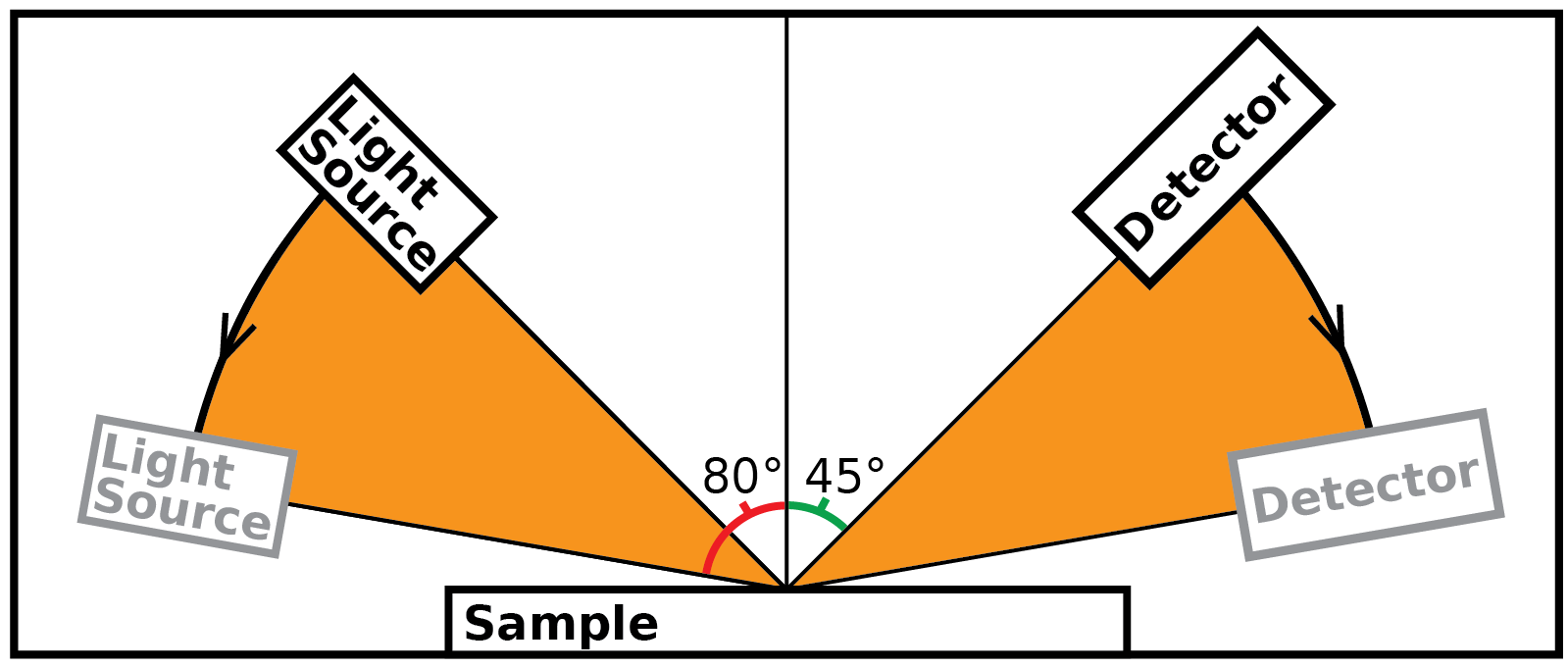


**Figure S1:** The VASE/VAR system can translate between many source and detection angles that are measured from the axis normal to the sample.

Variable-angle SE (VASE, J. A. Woollam) at angles between 45º and 75º in 5º increments were performed *ex situ* after deposition was complete and the native oxide layer had grown (1-2 nm). Both data sets were fit using a Drude-Lorentz model with one Drude and three Lorentz oscillators as described in reference.[^1–3^](https://paperpile.com/c/SssfIo/PvQz+NNIk+3PTP) The model used to fit the VASE measurements also included a TiO_x_ layer on the surface, and as a result of surface oxidation, the TiN was usually measured to be thinner than shown by the *in situ* measurements. The one exception is the ~20 nm (300 cycles) sample, which was more difficult to fit with the same parameters as the other samples likely because it was the first deposition performed in the series shown here and contained more contamination from the residual material in the PEALD tool. This trend has been discussed in previous work using the same tool.[^3^](https://paperpile.com/c/SssfIo/3PTP)


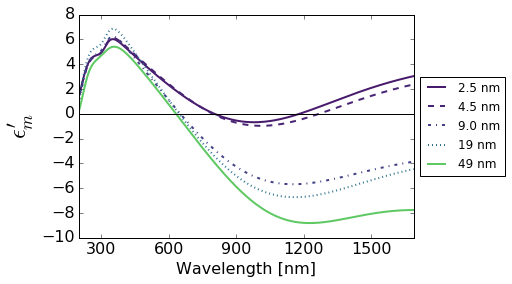

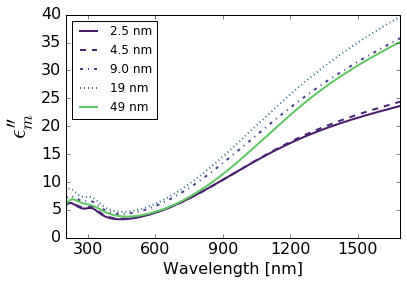


**Figure S2:** The as-deposited, *in situ* dielectric function measurements of TiN. Even the thinnest sample shows a small plasmonic window (where $\epsilon_{m}^{'}<0$) prior to film exposure to air. The as-deposited film thicknesses of 2.5 nm, 4.5 nm, 9.0 nm, 19 nm, and 49 nm correspond to depositions that were 55 cycles, 80 cycles, 150 cycles, 300 cycles, and 600 cycles. The 300-cycle film is unexpectedly measured to be thinner *in situ* than *ex situ*, which is likely due to more contamination during deposition decreasing the fit quality from the *in situ* data. The *in situ* root mean square error (MSE) for this film is 17 whereas for the 150-cycle and 600-cycle films it is 14 and 11, respectively. The *ex situ* VASE measurements have an extra degree of freedom (the variable angle) and so can provide a more accurate measurement (MSEs <13, **Figure S6a**).

In the case of the TiN films, all exhibited plasmonic regions as measured *in situ* (**Figure S2**), this plasmonic quality was no longer evident in the VASE measurements for the thinnest two films (**Figure 2a**) presumably due to the reduced thickness from the native oxide growth as well as possible oxidation between the nanocrystalline grain boundaries.[^4^](https://paperpile.com/c/SssfIo/JZaE) VA reflectometry (VAR) measurements were performed *ex situ* using the same VASE setup and measuring at angles between 45º and 80º in 5º increments to characterize the photonic crystal structures.

1. **Conductivity measurements of flat TiN films**

Conductivity of the flat TiN films was directly measured by the van der Pauw method (Ecopia HMS-5000) at room temperature and was calculated from a fit to a Drude-Lorentz model of SE and VASE data using $\sigma=\mu N_{e} e$ where σ is the DC conductivity of the material, μ is the mobility, *N_e_* is the carrier concentration, and *e* is the electron charge. The van der Pauw results, which are electrical measurements and strongly affected by grain boundary scattering, show lower TiN conductivity than both ellipsometric estimates, which are optical in nature and do not observe the grain boundary scattering of the carriers. The effect of the grain boundary scattering is more pronounced for thinner films or films with smaller grains. However, it should be noted that even the thinnest film (2 nm) exhibited high electrical conductivity (using the van der Pauw method) under ambient conditions showing good resistance to oxidation even after exposure to air, which causes surface oxidation. Corresponding inverse opal photonic crystals were fabricated during the same deposition runs, and so the same conductive properties should apply to the TiN conformal film throughout the inverse opal structure.

1. **Annealing effects on TiN films**

The anneals were performed with progressively increasing temperature since the added thermal energy and the reducing hydrogen gas introduce the irreversible effects of grain reordering as well as removal of contaminants. To verify that annealing is additive and previous lower temperature anneals minimally affect a later anneal at a higher temperature, other flat film samples which only experienced one anneal were also used, and they showed good agreement with the films that experienced all anneals. The measurements plotted in Figures 4 and S3-6 were from the films that only experienced one anneal. SiO_2_ skeletons coated with 150 cycles, 300 cycles, and 600 cycles of TiN were annealed simultaneously with their respective flat films and then measured with VAR. After each anneal (650ºC, 800ºC, 950ºC, and 1100ºC) their reflected intensities were recorded from 45º-80º in 5º increments.


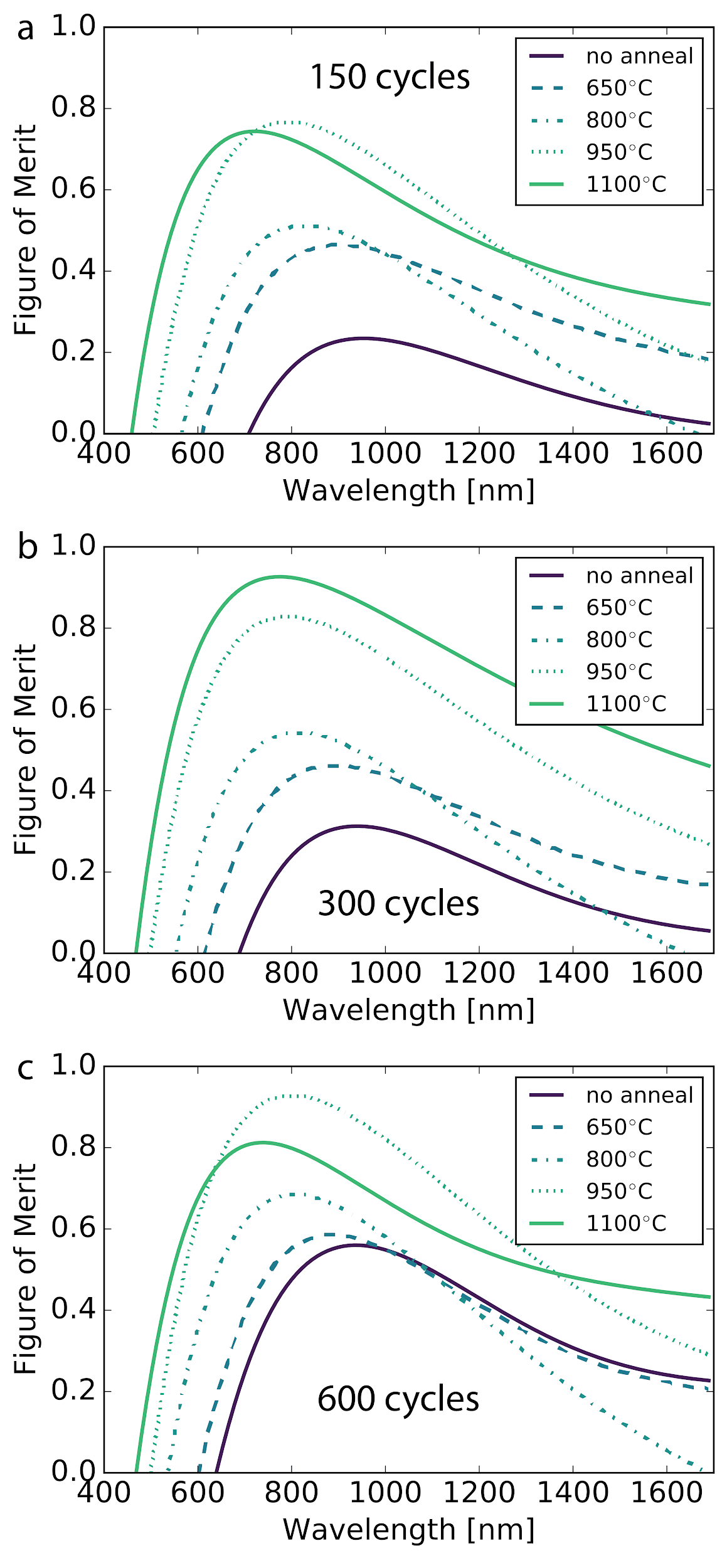


**Figure S3: Figures of merit vs. anneal temperature.** Annealing also causes a change in the plasmonic properties of the TiN. The figures of merit (FOMs) for each anneal condition and film thickness (a) 150 cycles, (b) 300 cycles, and (c) 600 cycles demonstrate the evolution in film plasmonic quality in the visible and near infrared.


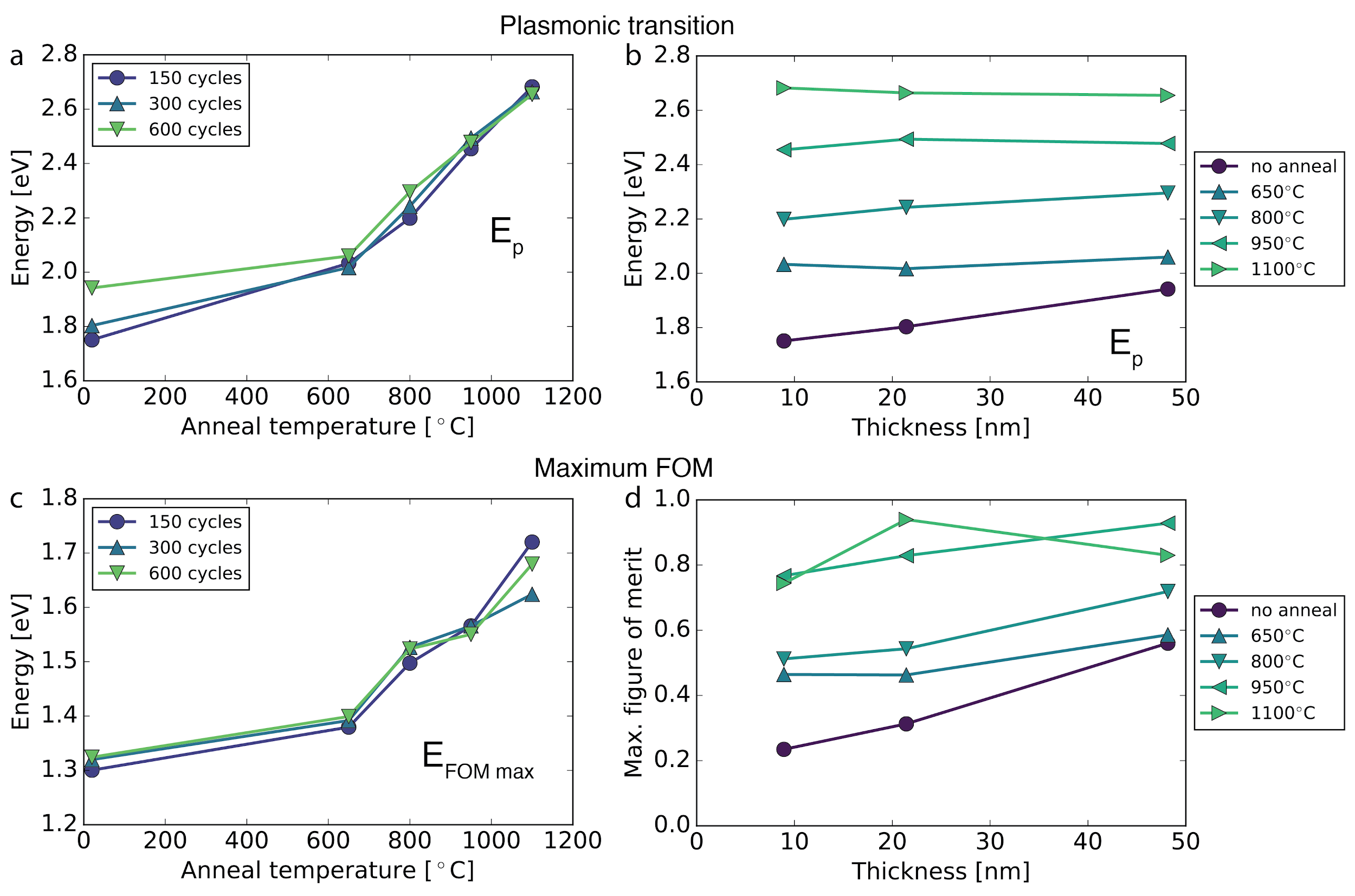


**Figure S4: Plasmonic properties vs. anneal temperature.** Extracting individual data points from the FOM plots is useful for understanding the plasmonic behavior of the films. (a,b) The films’ plasmonic transition energy (E_p_) blue-shifts with increasing anneal temperature and after anneals shows less dependence on the film thickness in contrast to the as-deposited samples. (c,d) The peak FOM also blue-shifts with increasing annealing temperature. Slight differences in film thicknesses versus the main text are due to differences in the ellipsometric fitting within the model’s error range.


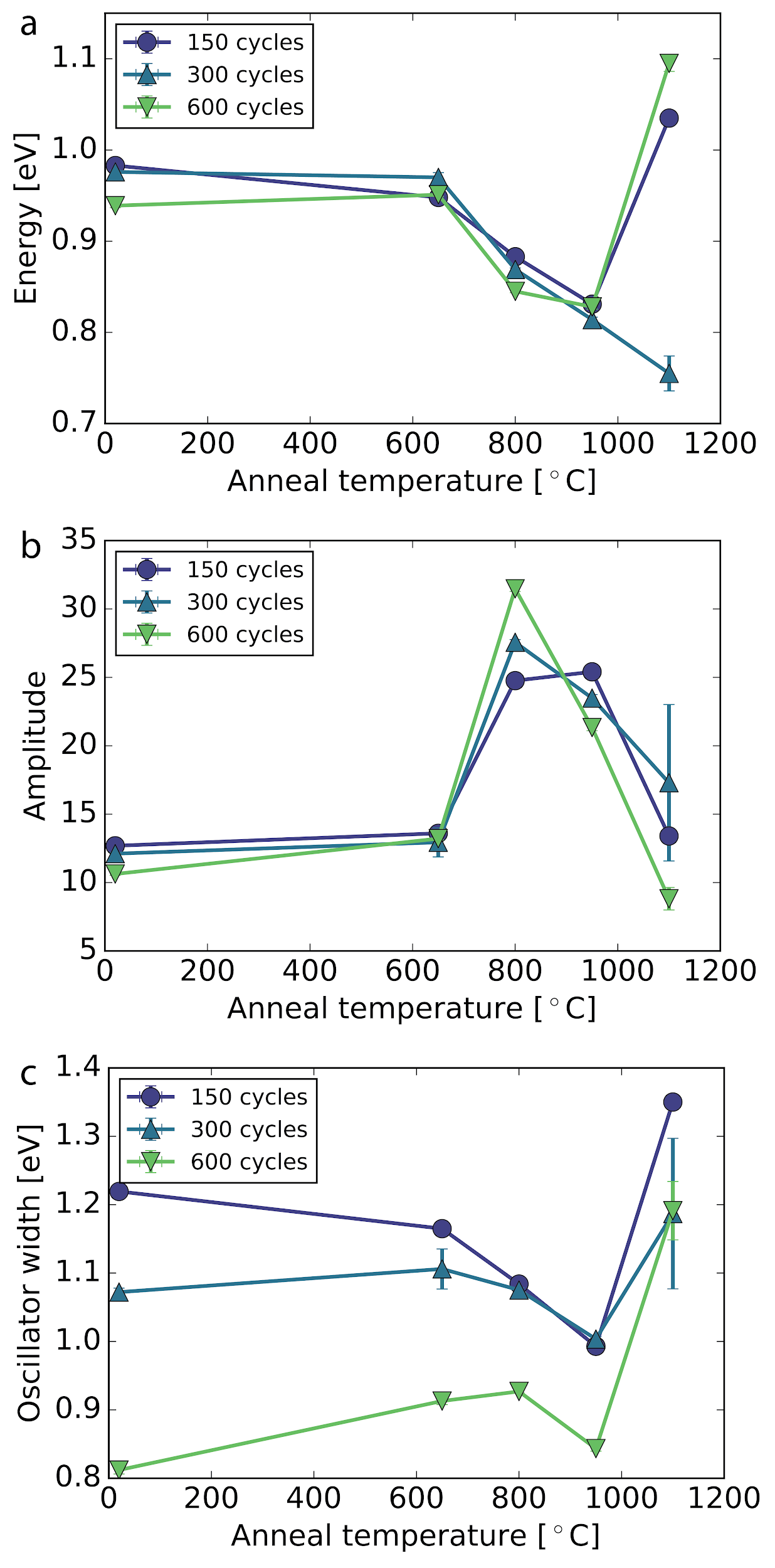


**Figure S5: Oscillator fit parameters vs. anneal temperature.** The lowest energy Lorentz oscillator added to the SE model is usually located ~1 eV, but its exact properties shift with annealing temperature. The (a) oscillator energy, (b) amplitude (dimensionless), and (c) oscillator width can describe the varying optical properties in the near infrared dependent on anneal temperature.

The 300-cycle sample annealed at 1100ºC exhibits behavior different than expected based on previous anneals and measurements due to the timing of its deposition. (This film was first in the series to be deposited in the shared PEALD tool following previous oxide deposition, and more cleaning or a TiN pre-deposition or conditioning could have helped the quality.[^3,5^](https://paperpile.com/c/SssfIo/3PTP+WXfs)) This is evident in high FOM in Figure S3 and the low energy position and the large error bars in the amplitude and width of the Lorentz oscillator in Figure S5. When fitting the VASE data, the software optimizes for a MSE, and is shown in Figure S4a for all samples. While most have acceptable MSE values of ~6-14, the MSE for the 300-cycle sample annealed at 1100ºC was unable to be optimized with an MSE below 20. The main contributing factor to this error is the carrier concentration (note the large error bar of 18% in Figure S4b), which determines the Drude parameter and is most evident in the ultraviolet. Figure S4c shows the SE fit of the raw measurement data where the fit is poorest below 400 nm.

**
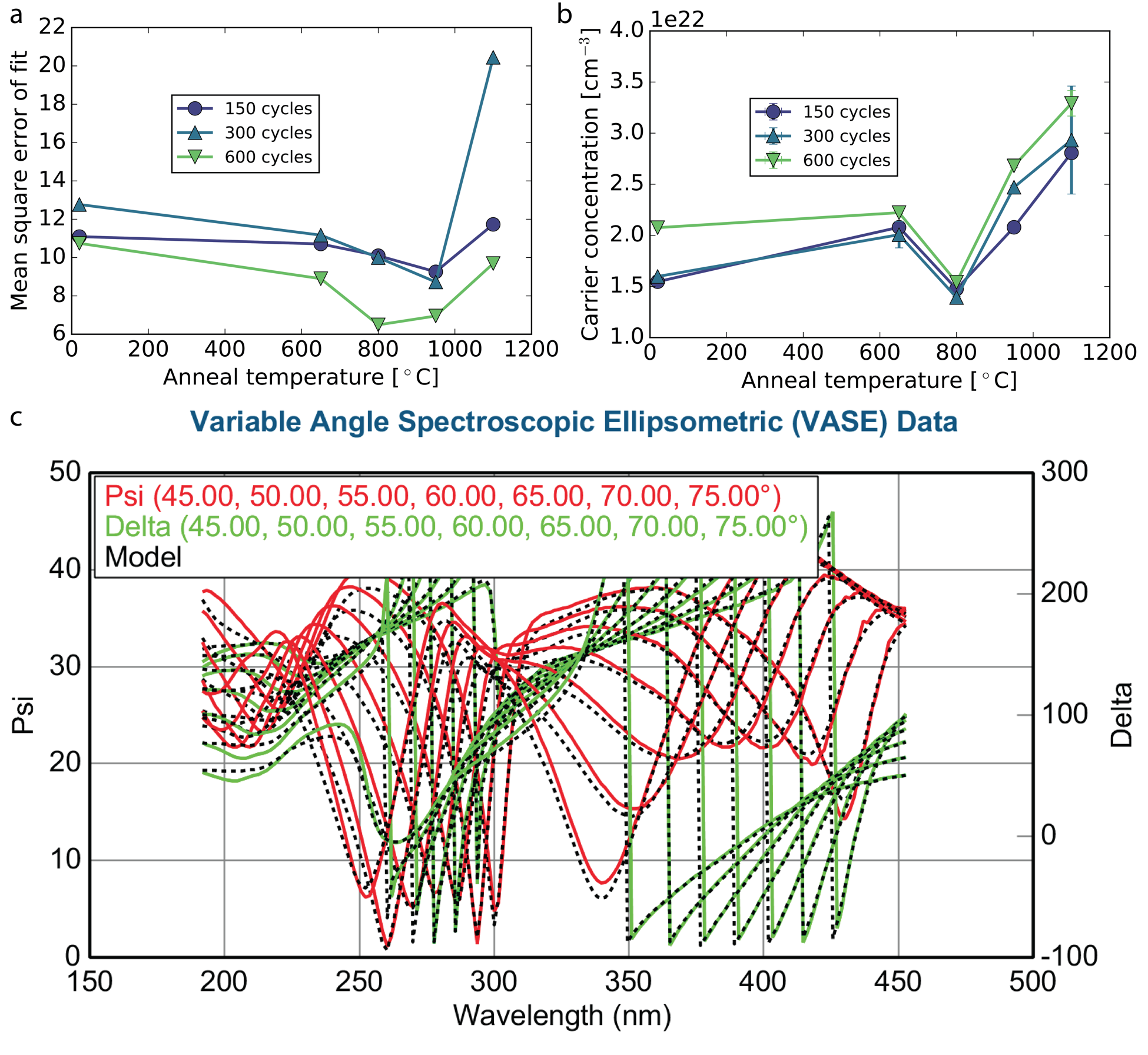
Figure S6: Carrier concentration and ellipsometry fit.** (a) The fit quality of the spectroscopic ellipsometry measurements can be quantified by the root mean square error (MSE) of the fit. All samples exhibit a reasonably good MSE except the 300-cycle sample after annealing at 1100ºC. (b) This high MSE (>20) is due to high error (18%) in the fitting of the carrier concentration of that sample. (c) For the spectral fits of this sample, the poor fit is reflected through a poor fit in the sub-400 nm wavelength region, or where the Drude parameter of the dielectric function is dominant.

1. **Annealing effects on inverse opals**


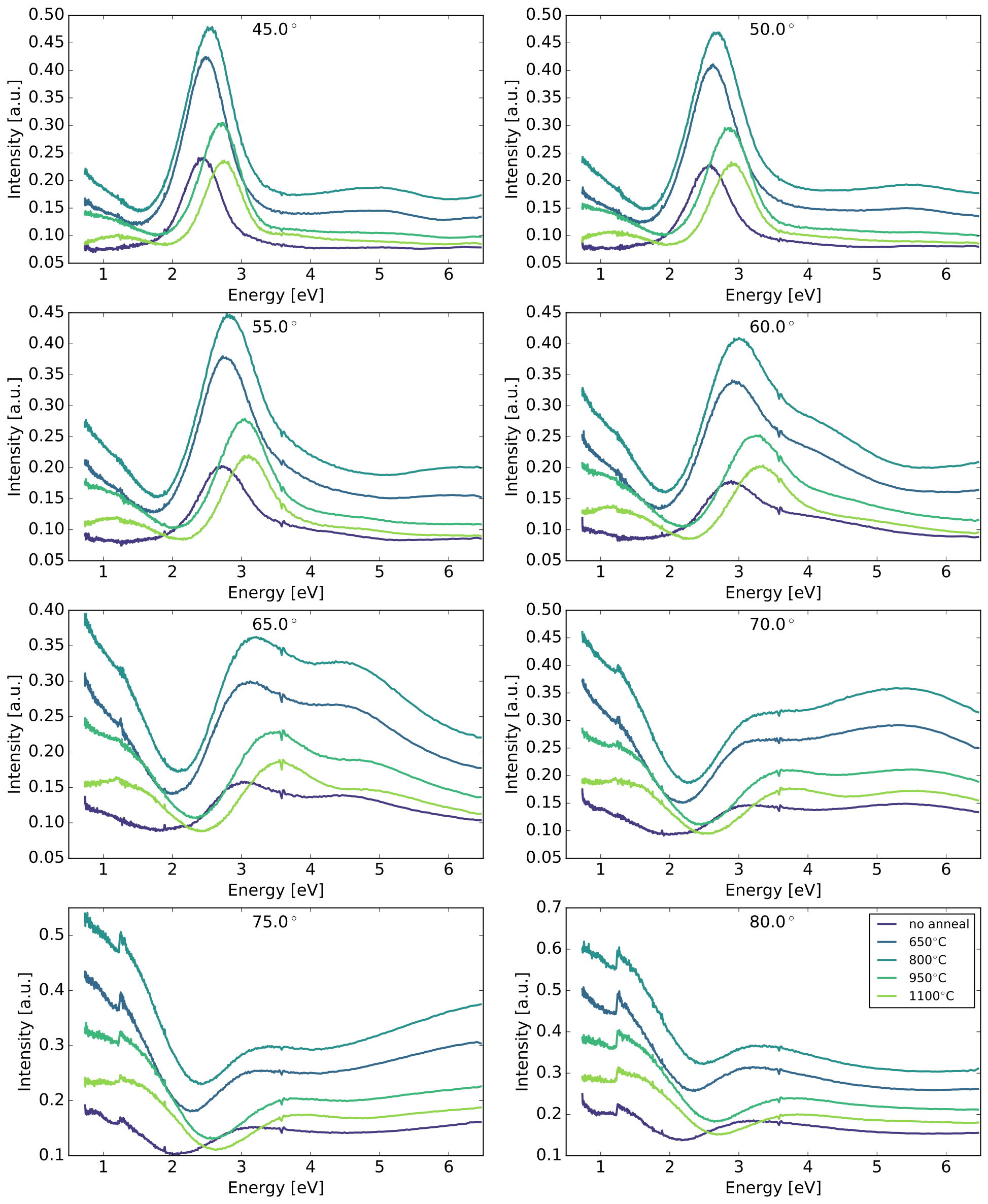


**Figure S7: Photonic crystal resonance shifts.** 150 cycles (7-10 nm) of TiN was deposited onto a SiO_2_ inverse opal lattice, measured with VASE at 8 angles (45º, 50º, 55º, 60º, 65º, 70º, 75º, 80º), annealed, measured, *etc.* It should be noted that the reflected intensity measurements from the inverse opals were normalized by the reflected intensity measured from a flat region of the sample. Annealing causes a blue shift in photonic crystal resonance, with the peak reflected intensity occurring after the 800ºC anneal.

**References**

1. [Langereis, E. *et al.* In situ spectroscopic ellipsometry as a versatile tool for studying atomic layer deposition. *J. Phys. D Appl. Phys.* **42**, 073001 (2009).](http://paperpile.com/b/SssfIo/PvQz)

2. [Langereis, E., Heil, S. B. S., van de Sanden, M. C. M. & Kessels, W. M. M. In situ spectroscopic ellipsometry study on the growth of ultrathin TiN films by plasma-assisted atomic layer deposition. *J. Appl. Phys.* **100**, 023534 (2006).](http://paperpile.com/b/SssfIo/NNIk)

3. [Otto, L. M. *et al.* Plasma-enhanced atomic layer deposition for plasmonic TiN. in *Nanophotonic Materials XIII* (2016). doi:](http://paperpile.com/b/SssfIo/3PTP)[10.1117/12.2238340](http://dx.doi.org/10.1117/12.2238340)[.](http://paperpile.com/b/SssfIo/3PTP)

4. [Miikkulainen, V., Leskelä, M., Ritala, M. & Puurunen, R. L. Crystallinity of inorganic films grown by atomic layer deposition: Overview and general trends. *J. Appl. Phys.* **113**, 021301 (2013).](http://paperpile.com/b/SssfIo/JZaE)

5. [Otto, L. M. Engineering Materials and Characterization Methods for Mass-Produced Plasmonic Devices. (University of Minnesota, 2017).](http://paperpile.com/b/SssfIo/WXfs)
